# Supplementary material for: SHaploseek is a sequencing-only, high-resolution method for comprehensive preimplantation genetic testing
Source: Sci Rep. 2023 Oct 21;13:18036. doi: 10.1038/s41598-023-45292-z (PMC10590366; doi:10.1038/s41598-023-45292-z)
Supplement: Supplementary file 1 — Supplementary Information 1. [file 41598_2023_45292_MOESM1_ESM.pdf]

Supplementary Materials and Methods

In this document, we describe our computational method for haplotype prediction in SHaploseek. The method is similar to that we have previously developed [2, 3], except that here we model the uncertainty in the genotype calls of the parents and other relatives using a model for the number of sequencing reads covering each allele. We divide the description of the method into two parts, depending on whether the other relatives that were sequenced (in addition to the parents) are grandparents or an affected child.

1 *Haplotype prediction with grandparental references*

We process each embryo independently using the method described below. For each embryo, our data includes low-coverage sequencing reads from the embryo biopsy and low-coverage bulk sequencing reads for both parents and for at least one grandparent.

Sites were excluded from haplotype prediction if:

1. The variant was an indel. I.e., we only considered single-nucleotide variants (SNVs).
2. The variant was called in the parents and all other relatives as homozygous reference.
3. The allele frequency of the SNV in non-Finnish Europeans based on gnomAD was less than 10%.
4. The number of reads covering the SNV for any parent or grandparent was more than 4 times the average sequencing depth for parents and grandparents, or the number of reads covering the SNV for any embryo was more than 8 times the average sequencing depth for embryos.
5. The SNV was not covered by a single read in any embryo.

We then run a hidden Markov model (HMM) on the remaining sites. The HMM then infers a hidden inheritance state at each of those SNVs. To do so, we must define the states, emissions probabilities, and transition probabilities of the HMM.

1.1 Hidden states

There are four possible hidden inheritance states at a given SNV:

1. The embryo received the two haplotypes that were maternally transmitted to each of the embryo's parents.
2. The embryo received the two haplotypes that were paternally transmitted to each of the embryo's parents.
3. The embryo received the haplotype that was maternally transmitted to the embryo's mother and the haplotype that was paternally transmitted to the embryo's father.
4. The embryo received the haplotype that was paternally transmitted to the embryo's mother and the haplotype that was maternally transmitted to the embryo's father.

1.2 Emissions probabilities

At a given SNV, let the total number of reads observed for individual j be t_j and let the number of reference reads observed for individual j (embryo, father, mother, or grandparent) be r_j. The three possible unphased genotypes at a particular SNV are AA, AB and BB. We start by calculating the three probabilities $P(r_j|t_j, AA)$, $P(r_j|t_j, AB)$ and $P(r_j|t_j, BB)$ for each individual at each SNV. If individual j has no reads observed at the SNV (either sporadically or if no sequencing data is available for a grandparent), we set these three probabilities equal to 1. Otherwise, these probabilities are calculated using the beta-binomial distribution.

To estimate the parameters of the beta-binomial distribution, we used paired sequencing and microarray data for the same individuals from Family 1, where we assumed the microarray data is error-free. The beta-binomial distribution has two parameters (for a given number of trials t_j), a success probability (the probability an allele is the reference allele) and an overdispersion parameter. For low-coverage single-cell sequencing data, we estimated a different success probability for each true genotype and a common over-dispersion parameter for the three true genotypes; for bulk sequencing data we estimated different

success probabilities and over-dispersion parameters for each of the three true genotypes. We estimated the parameters using maximum likelihood estimation.

We label the maternal grandmother, maternal grandfather, paternal grandmother, and paternal grandfather with the subscripts mgm, mgf, pgm and pgf, respectively. We label the mother, father, and embryo with the subscripts m, f and e, respectively.

Let the unphased genotype of individual j (at a given SNV) be g_j. For each combination of unphased genotypes of the seven family members (four grandparents, two parents, and the embryo) we calculate the probability of these genotypes given the inheritance state s,

$$P(g_e, g_m, g_f, g_{mgm}, g_{mgf}, g_{pgm}, g_{pgf}|s) = \\ P(g_e, g_m, g_f|g_{mgm}, g_{mgf}, g_{pgm}, g_{pgf}, s)P(g_{mgm}, g_{mgf}, g_{pgm}, g_{pgf}) \quad (1)$$

Above, we used $P(g_{mgm}, g_{mgf}, g_{pgm}, g_{pgf}) = 1/3^4$ for each of the 3^4 possible four grandparental (unphased) genotypes. For the conditional probability $P(g_e, g_m, g_f|g_{mgm}, g_{mgf}, g_{pgm}, g_{pgf}, s)$, we first computed the corresponding probability using *phased* genotypes. Given a heterozygous genotype (AB), we assumed that both phased genotypes (AB or BA) are equally likely. We then used the laws of Mendelian inheritance to compute the probability of each combination of parental and embryo (phased) genotypes given the grandparental (phased) genotypes and the inheritance state. Impossible combinations (for example, all grandparents are homozygous for the reference allele and both parents and the child are homozygous for the alternate allele) were given probability zero. Finally, we computed the probability for each *unphased* heterozygous genotype as the sum over the probabilities of the pair of corresponding phased genotypes.

Finally, let the set of all possible combinations of genotypes for the seven individuals be G. Let the set of the seven individuals be J. Our emissions probabilities for each of the four inheritance states at a particular SNV are then given by the sum

$$\sum_G \left\{ P(g_e, g_m, g_f, g_{mgm}, g_{mgf}, g_{pgm}, g_{pgf}|s) \left[\prod_{j \in J} P(r_j|t_j, g_j) \right] \right\}. \quad (2)$$

1.3 Transition probabilities

For the transition probabilities in our HMM, we used the sex-specific recombination rates from Bhérer et al. (2017) [1].

1.4 Inference

We used the forward-backward algorithm to calculate the marginal posterior probability of each hidden inheritance state at each SNV. We used the Viterbi algorithm to calculate the most likely sequence of hidden inheritance states at all SNVs. If no grandparents are available for one of the parents, then all posterior probabilities for the haplotype that was transmitted to the embryo from that parent will be 50/50 for the haplotype to descend from the paternal/maternal grandparent.

2 Haplotype prediction with a child reference

Our computational method for haplotype prediction using a child reference sample is similar to that described in Backenroth et al. (2019) [2]. Here, we used the beta-binomial distribution (as described in Section 1) to calculate the probability of a particular configuration of parental or reference child sequencing reads, given an assumed parental or reference child genotype.

References

[1] C. Bhérer, C. L. Campbell, and A. Auton. Refined genetic maps reveal sexual dimorphism in human meiotic recombination at multiple scales. Nature Communications 8, 14994 (2017).

[2] D. Backenroth, F. Zahdeh, Y. Kling, et al. Haploseek: A 24-hour all-in-one method for preimplantation genetic diagnosis (PGD) of monogenic disease and aneuploidy. Genetics in Medicine 21, 1390 (2019).

[3] D. A. Zeevi, D. Backenroth, E. Hakam-Spector, et al. Expanded clinical validation of Haploseek for comprehensive preimplantation genetic testing. Genetics in Medicine 23, 1334 (2021).
